# Elucidating Sexual and Spatial Influences on the Trophic Ecology of Amazon River Dolphins (*Inia geoffrensis*) and Mercury Contamination

**DOI:** 10.1021/acs.est.5c07115

**Published:** 2025-10-16

**Authors:** Monizze Vannuci-Silva, Vera Maria Ferreira da Silva, Lucas Rodrigues Tovar, Rodrigo de Souza Amaral, Bárbara M. R. Manhães, Gleici Montanini, Alexandre de Freitas Azevedo, José Lailson-Brito, Tatiana Lemos Bisi

**Affiliations:** † Laboratório de Mamíferos Aquáticos e Bioindicadores, Faculdade de Oceanografia, 28130 Universidade do Estado do Rio de Janeiro (UERJ), Rio de Janeiro 20550-013, Brazil; ‡ Programa de Pós-Graduação em Oceanografia, Faculdade de Oceanografia, Universidade do Estado do Rio de Janeiro (UERJ), Rio de Janeiro 20550-013, Brazil; § Laboratório de Mamíferos Aquáticos, 191073 Instituto Nacional de Pesquisas da Amazônia (INPA), Manaus 69067-375, Brazil; ∥ Instituto Federal de Educação, 119525 Ciência e Tecnologia do Amazonas (IFAM). Campus Manaus Zona Leste, Manaus 69086-475, Brazil

**Keywords:** floodplain, Inia geoffrensis, stable isotopes, mercury exposure, niche overlap, Amazon droughts

## Abstract

The Amazon Basin hosts an immense biodiversity, including the endemic Amazon River dolphin (*Inia geoffrensis*). Dietary exposure to mercury (Hg) is one of the threats to this top predator species. This study investigated the relationship between Hg exposure, trophic position and foraging areas in *I. geoffrensis*. We analyzed Hg concentrations and stable carbon (δ^13^C) and nitrogen (δ^15^N) isotope ratios in 111 blood samples collected in different years. The Hg concentrations ranged from 23 to 582 μg/kg wet weight, while mean δ^13^C and δ^15^N values were −29.6 ± 1.7‰ and 9.8 ± 0.5‰, respectively. Narrower isotopic niche areas were observed in the latest year analyzed, suggesting reduced prey availability and/or restricted foraging areas. Males displayed greater niche plasticity, whereas females maintained consistent feeding strategies over time. Spatial segregation between males and females/juveniles appeared to be the main driver of differences in Hg concentrations. These findings raise concerns for species conservation under the increasing frequency of extreme Amazon droughts, which can alter Hg dynamics and degrade habitats. Monitoring *I. geoffrensis* health is essential not only for the protection of this endangered species, but also as a sentinel for Hg exposure risk in the Amazonian human population, mainly riverine and indigenous communities.

## Graphical abstract

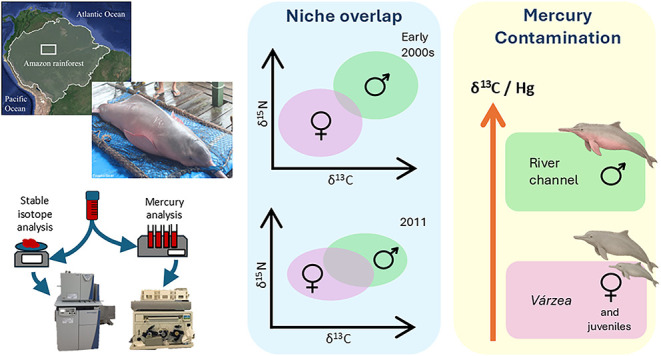

## 1 Introduction

Floodplain areas in the Amazon forest, known as *várzea*, have high biological productivity and biodiversity due to the variety of environments and the high levels of nutrients in its waters, playing an important role in the Amazon aquatic and terrestrial ecosystems.^1,2^ This globally important region has a historical relationship with gold mining activity, which makes the Amazon Basin a unique area for ecotoxicological studies of trace elements.^3–5^ The gold rush was one of the most environmentally destructive activities in the region. Mercury (Hg) emissions were estimated at around 2,000 tons from 1979 to 1996.^5^ Exposure continues due to illegal activities of artisanal small-scale gold mining (ASGM), which still occur in the area. Elevated Hg concentrations detected in hair samples from Yanomami indigenous villages have been associated with the proximity to ASGM sites,^6,7^ while risks associated with Hg exposure through fish consumption have also been reported.^8^ Wildfires, in addition to destroying the biome, cause high Hg emissions into the atmosphere and leaching of this toxic element into aquatic ecosystems.^9,10^ Furthermore, the region is currently exposed to new sources of pollutants due to the increase in human population in the cities, extensive agricultural exploitation of its land, and major works in operation and/or underway, such as constructing of numerous hydroelectric dams and ports.^11^ The scenario remains alarming for the future, as climate change may alter the bioavailability of contaminants, disrupt the flood pulse, and cause significant habitat degradation.^12–15^

Several aquatic endemic species inhabit the *várzea*, including many endangered ones, such as the Amazon River dolphin (*Inia geoffrensis*).^16^ It is the largest river dolphin, is a predominantly piscivorous species with solitary habits, and is strongly associated with the banks of water bodies.^16,17^ Its geographic distribution constitutes the main rivers, tributaries, and lakes of the Amazon Basin.^16^ *I. geoffrensis* is classified as “Endangered” by the Red List of the International Union for Conservation of Nature (IUCN). Pollution, habitat loss, and fragmentation are the main threats to the species caused by human activities.^18,19^ Anthropogenic pressure on the Amazon basin has led to a 70.4% decrease in the *I. geoffrensis* population over the past 22 years.^20^

Odontocete cetaceans, such as *I. geoffrensis*, are considered monitors of the quality of aquatic systems, quantifying the contaminants’ bioavailability in these ecosystems due to their ability to accumulate chemicals, long life span, and high trophic position.^21,22^ They can integrate spatial and temporal variations in the contamination of an environment and quantify the ecological significance of contamination.^21,23–25^ Studies on Hg in *I. geoffrensis* in the literature reported concentrations in milk, kidney, liver, muscle, and adipose tissue.^23,26–28^ Those Hg concentrations are commonly high and suggest that the Hg bioaccumulation process is active in these dolphins. Indeed, the levels found in their milk approach the toxicity threshold of methylmercury for humans (200 μg mercury per liter) and indicate maternal transfer of this pollutant.^28^

Given the exposure scenario and the high trophic level occupied by the species, monitoring Hg levels is essential. Due to the challenges of obtaining fresh tissues from deceased animals, nonlethal sampling methods appear more suitable for monitoring programs. Blood samples meet this criterion as they offer insights into recent contamination and reflect the distribution of absorbed contaminants throughout the organism.^29^

Considering that Hg exposure in aquatic mammals is mainly through diet,^30^ analyzing carbon and nitrogen stable isotopes is a relevant tool in ecotoxicological studies, providing information on the organisms’ foraging area, diet, and trophic level.^31,32^ The Hg biomagnification was already evidenced by the increased concentration of this element along the food web. Variations due to spatiotemporal changes in the diet and the complexity of the food web, associated with environmental changes and impacts, may play a role in Hg accumulation in top predators of the Amazon river systems.^33^ Many studies have used carbon and nitrogen stable isotopes as trophic level indicators to investigate the transfer of contaminants and energy through food webs.^34–36^ Besides that, stable isotopes can be used to compare trophic relationships between individuals of the same species with, for example, different foraging areas, different stages of development, sex, and opportunistic foraging habits.^33^ These possibilities of the technique are particularly interesting to investigate Hg accumulation in species that exhibit intraspecific ecological segregation, as already reported for *I. geoffrensis*.^37^

The objectives of this study were (1) to evaluate the Hg contamination and its relationship with biological, ecological, and temporal factors in *I. geoffrensis* and (2) to investigate intraspecific resource partition in these animals. For this purpose, Hg concentrations were determined in blood samples, and carbon and nitrogen stable isotope measurements were performed to assess trophic ecology.

## 2 Materials and Methods

### 2.1 Study Area

Amazonian rivers experience flood pulses, and, during flood periods, their waters occupy floodplains (*várzeas*) and floodforests (*igapós*). In this way, rivers contribute to the transfer of dissolved substances, including nutrients and pollutants, and sediments between the main channel and the floodplain, and these processes are associated with the influence of hydrological regimes.^38,39^ The rivers of the Amazon basin vary considerably in their physical and chemical composition due to their different environments. Amazonian waters are classified into three main types: clear, white, and black water rivers.^39^ White water rivers are those of Andean and sub-Andean origin and have turbid, muddy, and yellowish waters. These features are due to erosive processes along their banks, which increase the amount of particulate matter in suspension. Among the white water rivers in the Amazon basin are the Solimões and the Japurá rivers.^39^

Samples were collected in the Japurá River within the Mamirauá Sustainable Development Reserve (MSDR). The Solimões and the Japurá rivers converge in the MSDR, located in the Central Brazilian Amazon and encompassing an area of 11,240 km^2^, which is the largest Amazonian *várzea* reserve^40^ (Figure 1). It is located within a floodplain, or *várzea*, with water levels rising to 15 m during the wet period.^41^

**Figure 1:**
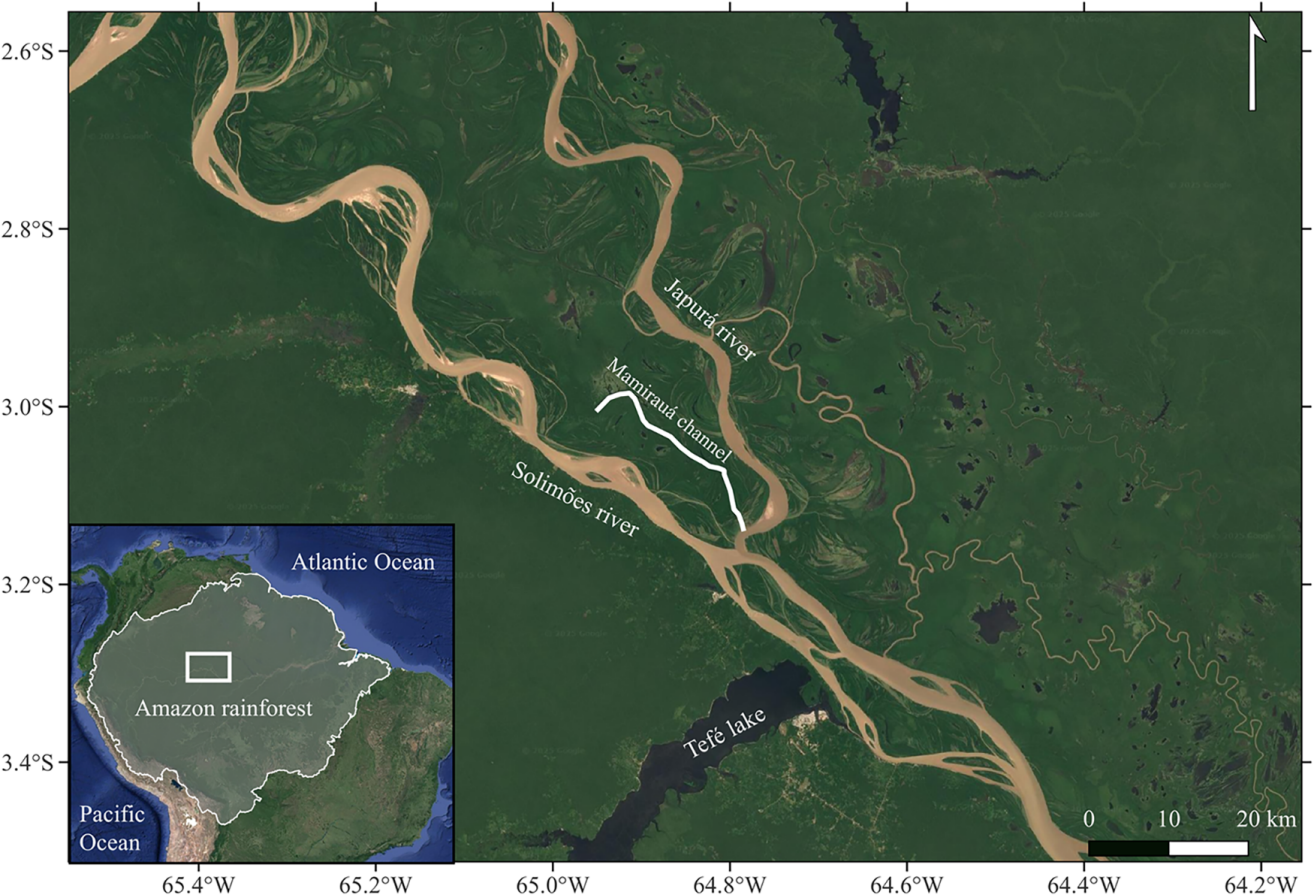
Map of the study area, the Japurá River and the Mamirauá Sustainable Development Reserve (MSDR), Central Brazilian Amazon.

### 2.2 Sampling

The whole blood samples of *I. geoffrensis* were collected from wild and live animals that inhabit the interior or surroundings of the MSDR in the years 2002 to 2006 and 2011. A total of 48 females and 63 males were sampled by the “Projeto Boto” from the Aquatic Mammal Laboratory of the National Institute for Amazonian Research (INPA). The “Projeto Boto” captures live botos for tagging, biological material collection and monitoring at the MSDR. Biological parameters—sex, length (cm), and weight (kg)—were also collected. All samplings were carried out with authorizations granted by IBAMA/SISBIO (National Environmental Agency) under numbers 13462-1 to 4. The dolphins were captured using a refined seining method described by da Silva and Martin^42^ and then guided to a shallow shoreline before being transferred by boat to a nearby floating laboratory. Briefly, a net was deployed to fully block the river channel. A secondary small-mesh net was then used to herd the animals toward a gently sloping shore. Individuals showing any external indications of ill health were excluded from further analyses.^42^

Age classes were determined following the criteria established by Martin and da Silva:^17^ adults were identified by reaching an asymptotic body length and weight, and by the presence of widespread tooth-rake scars. Additionally, adult males often exhibited a distinctive pink coloration, and some also developed skin lesions.^17^ Calves were defined as individuals still nursing, while juveniles were young, independent individuals. The juvenile stage ends between 8 and 12 years of age, with males reaching a length of approximately 2 m and females up to 1.75 m. Males longer than 2 m and females longer than 1.8 m were classified as adults.^17^

Blood samples of approximately 15 mL were collected from the caudal peduncle vein from *I. geoffrensis* restrained at the floating research station using sterile syringes and needles as described by Mello & da Silva.^43^ After sampling, the whole blood was frozen and sent to the Laboratory of Aquatic Mammals and Bioindicators (MAQUA) at the State University of Rio de Janeiro (UERJ), where total mercury (THg) determination and stable isotope measurements were carried out. Samples were homogenized using an Ultra-Turrax disperser (T25 digital, IKA, Germany) for 2 min at a speed of 8 rpm, and aliquots were separated as follows: 0.7 g for THg; while approximately 0.8 to 1.0 g of samples were dried and macerated for stable isotope analysis.

### 2.3 Total Mercury (THg) Determination

Total mercury determination was adapted from procedures described in the literature.^44^ Briefly, 0.7 g of wet whole blood sample was weighed, and 5 mL of a mixture of sulfuric and nitric acid (HNO_3_/H_2_SO_4_, 1:1) was added. After that, 1.5 mL of hydrogen peroxide (H_2_O_2_) was added, and the solution was heated to 60 °C for 2 h 30 min until complete solubilization. After this digestion step, 5 mL of potassium permanganate was added, and the mixture was heated again for 15 min at 60 °C. The samples were then kept overnight in this oxidizing medium to ensure Hg remained in solution until analysis on the following day. Prior to measurement, hydroxylamine was added to reduce the excess oxidizing agent and release Hg from its oxidized state. THg concentrations were determined by Cold Vapor/Atomic Absorption (FIMS-400, PerkinElmer, USA) with sodium borohydride as a reducing agent. The accuracy and precision of the analytical method were verified using certified reference materials (CRM) from the National Research Council (NRC), Canada, and results were in good agreement with certified values (96 ± 4% for DORM-2 and 98 ± 2% for DOLT-5). The limit of detection (LOD) of the equipment was 0.006 μg/L and the limit of quantification (LOQ) was 5 μg/kg. Quality assurance was also guaranteed by the analysis of procedural blanks (0.043 ± 0.006 μg/L) and sample replicates (coefficient of variation < 20%). Concentrations were expressed in μg/kg, wet weight.

### 2.4 Stable Isotope Measurements

All samples were dried in an oven at 60 °C for 72 h and then ground into a homogeneous powder with a mortar and pestle. Aliquots of 0.3 mg dried whole blood samples were weighed into tin capsules. An Isotope Ratio Mass Spectrometer (IRMS-Delta V Advantage, Thermo Fisher Scientific, USA) coupled to a C–N–S elemental analyzer (Flash EA 2000, Thermo Fisher Scientific, USA) was used for the measurements of carbon and nitrogen isotopic ratios. Stable isotope ratios were expressed in delta notation (δ) in parts per thousand (‰), using the formula δ*X* = [(*R*_sample_/*R*_standard_) – 1] × 1000, where *X* is ^13^C or ^15^N, and *R* represents the ratio ^13^C/^12^C or ^15^N/^14^N. The fossil Belemnite from the PeeDee formation - PDB and atmospheric N_2_ were used as standards for carbon and nitrogen, respectively. International isotope secondary standards (USGS24 and IAEA-N-2) and an internal standard were analyzed within each batch to ensure the precision and accuracy of the analytical method. The analytical precision was <0.2‰ for both δ^13^C and δ^15^N. No lipid normalization was applied once all samples showed C/N ratios below 3.4.^45^

### 2.5 Statistical Analysis

Statistical analyses were performed using the R software (The R Foundation for Statistical Computing, Vienna, Austria, 2019). For Hg concentrations, nonparametric tests were applied since the data did not show a normal distribution (Shapiro-Wilk, *p* < 0.05). The Mann–Whitney U test was used to compare concentrations between sexes and age classes, and the Kruskal–Wallis’ test was used to compare differences between sampling periods. Variations in δ^13^C and δ^15^N values between sampling years, sex, and age classes were investigated using analysis of variance (ANOVA) and the residuals were tested for normality and homogeneity of variances using the Kolmogorov–Smirnov and Levene tests, respectively.

The niche overlap examination between the years for both sexes was made with Kernel Utilization Density (KUD) estimators through the NicheRover R package. Niche width was compared between sex and years when more than 10 samples were available, and its differences were investigated by the Kruskal–Wallis’ test and Dunn test as a posthoc. KUD brings more richness for overlap estimation by including data density and data distribution over the bidimensional isospace, considering 50% and 75% data bandwidths.

A Generalized Linear Model (GLM) was used to understand the variations in THg concentrations and stable isotope values. The variables sampling year, sex, total length, and δ^13^C and δ^15^N values were tested. The best-fitting model was chosen based on the lowest Akaike information criterion (AIC) value, and the pseudo-R-squared was calculated by the McFadden method. The level of significance was 95% (*p* < 0.05).

For both KUD and GLM analyses, only immature and adult individuals were considered. Calves and juveniles were excluded as they could bias the data due to breastfeeding.^46^ All the data collected in 2002, 2003, 2006, and the data from females in 2005 were not included in the KUD analyses due to the insufficient sample size.

## 3 Results and Discussion

### 3.1 Mercury Concentrations

The THg concentrations are presented in Table 1. Except for data from 2006, the highest medians were reported for males, with concentrations ranging from 34 to 582 μg/kg, whereas females presented THg levels between 18 and 576 μg/kg. To the best of our knowledge, only one study has analyzed elements in the blood of a female *I. geoffrensis* using wavelength-dispersive X-ray fluorescence spectrometry.^47^ Although the survey detected and quantified 13 elements, Hg and other potentially toxic elements were not identified due to the detection limitations of the technique.

**Table 1:** Total Mercury Concentrations (THg, μg/kg Wet Weight) in Blood Samples of Amazon River Dolphin (*Inia geoffrensis*) Collected between 2004 and 2006 and 2011 in the Area of Mamirauá Sustainable Development Reserve, Brazilian Amazon

THg (μg/kg, wet weight)													
		Adult				Immature				All			
Year	Sex	*N*	Mean ± SD	Median	Min/Max	*N*	Mean ± SD	Median	Min/Max	*N*	Mean ± SD	Median	Min/Max
2002	Female	1	-	-	139	0	na	na	na	1	-	-	139
	Male	1	-	-	103	0	na	na	na	1	-	-	103
2003	Female	0	na	na	na	0	na	na	na	0	na	na	na
	Male	2	190 ± 78	190	135/245	0	na	na	na	2	190 ± 78	190	135/245
2004	Female	17	181 ± 107	137	76/476	2	74 ± 20	74	59/88	19	169 ± 107	132	59/476
	Male	14	222 ± 145	187	92/582	2	78 ± 37	78	52/104	16	204 ± 144	161	52/582
2005	Female	5	162 ± 154	87	54/426	1	18	18	18	6	138 ± 150	81	18/426
	Male	18	189 ± 74	178	50/377	4	75 ± 60	49	38/164	22	168 ± 83	162	38/377
2006	Female	6	165 ± 139	112	36/356	0	na	na	na	6	165 ± 139	112	36/356
	Male	4	108 ± 75	97	34/204	1	75	75	75	5	101 ± 66	75	34/204
2011	Female	13	190 ± 142	129	53/576	3	72 ± 78	31	23/162	16	168 ± 139	128	23/576
	Male	9	194 ± 89	204	83/358	8	125 ± 51	134	47/215	17	161 ± 80	143	47/358

Several studies have reported blood concentrations of THg in marine mammals. For example, Das et al.^48^ found THg levels in the blood of harbor seals (*Phoca vitulina*) ranging from 40 to 560 μg/kg. Numerous studies of bottlenose dolphins (*Tursiops truncatus*) from Florida reported THg concentrations in blood varying between 110 and 2570 μg/kg.^49–52^ The THg concentrations observed in the present study fall within this previously reported range (Table 1).

The Hg concentrations detected in blood in the present study are comparable to those reported by Rosas & Lehti^28^ (176 μg/L) in the milk of an *I. geoffrensis* specimen near the city of Manaus. Lailson-Brito et al.^3^ found Hg concentrations ranging from 90 to 36,000 μg/kg, wet weight, in the kidney and liver of four *I. geoffrensis* specimens from the Japurá River. Similarly, Mosquera-Guerra et al.^27^ reported a maximum concentration of 2600 μg/kg, wet weight, in the muscle of *I. geoffrensis* from different rivers of the Amazon Basin. Barbosa et al.^26^ reported concentrations of THg ranging from 15 to 3804 μg/kg in the adipose tissue of species of the genus *Inia* collected from the Madeira River Basin. As expected, blood Hg concentrations were lower than those found in organs that accumulate Hg,^34^ such as the liver, kidney, and muscle. The observed differences in Hg concentrations between blood and mercury-accumulating tissues highlight the role of blood as an indicator of recent Hg exposure rather than long-term bioaccumulation, due to its rapid turnover.^36^ Nevertheless, blood analysis remains an important tool for assessing exposure levels and recent contamination events.

The complex hydrological network of the Amazon River basin can transport pollutants far from their original sources, reaching protected areas such as the MDSR. The Caquetá River, which originates in Colombia and flows upstream of the Japurá River—the sampling area of this study—has been contaminated with Hg primarily due to illegal gold mining activities.^53^ Hydroelectric dams can also alter the Hg cycle by promoting deforestation, water stratification, and conditions that favor Hg methylation.^54^ As a result, Hg becomes more bioavailable and can be incorporated into the food web,^30,55^ even within conservation units, as corroborated by the Hg levels found in *I. geoffrensis* blood.

The concentration of THg in blood reflects the most recent dietary intake rather than long-term body burden.^36^ In aquatic mammals, including *I. geoffrensis*, the primary pathway of Hg exposure is through the ingestion of contaminated prey, making diet the main Hg source.^30^ The species exhibits a diverse diet encompassing over 40 fish species, with its heterodont dentition allowing predation on turtles and crabs as well.^16^ It is more generalist during the flood period, when the fish are more dispersed throughout the flooded forest, making it difficult to catch them. In the dry season, there is a higher density of fish, which are concentrated in the main river channels, leading to greater prey selectivity at this point in the water cycle.^16,37^ They prey mainly on piscivorous species,^16^ highlighting the high trophic position that the species occupies in this aquatic system. Indeed, even when analyzing samples of fast turnover, such as blood, Hg concentrations were detected in all the samples, including young (immature) individuals (Table 1). This result reflects the high Hg concentrations to which these animals are exposed daily in their diet, i.e., large fish from the region.

No significant differences were observed between the sampling periods (Kruskal–Wallis , *p* > 0.05). Significant differences between THg concentrations in males and females were observed only in 2004. These results may be related to the difference in feeding ecology between the sexes, resulting in greater Hg intake by males. This difference is discussed in the next section.

Cause-and-effect relationships between Hg bioaccumulation and aquatic mammal health are scarce in the literature^56^ due to ethical, logistical, and financial difficulties. The only study on *I. geoffrensis* suggested an indirect effect of Hg on greater susceptibility to pulmonary embolism.^57^ This effect, according to the authors, occurs due to reduced bioavailability of selenium, which is an essential antioxidant element and acts in the Hg detoxification complex.^58^ The fatty emboli may potentially become trapped in the pulmonary capillaries, ultimately leading to death.^57^ Unfortunately, the authors did not report the Hg concentrations in the tissues or other matrices where this adverse effect was reported. Regarding cetaceans in general, only one study in the literature has associated histological changes in the liver of *Tursiops truncatus* with Hg concentrations above 61,000 μg/kg wet weight.^59^

Considering marine mammals, some studies have demonstrated the immunotoxic potential of Hg through *in vitro* analysis of blood cells. Mercury concentrations above 1 μM impaired lymphocyte activity, proliferation, and survival in harbor seals blood cells and reduced gene expression in these exposed cells. The THg was assumed to be methylmercury (MeHg), and the immunosuppressive effects were attributed to this organic form of Hg.^48^ Similarly, Pellissó et al.^60^ reported a significant decrease in the lymphoproliferative response of *in vitro* leukocytes from captive bottlenose dolphins in Spain exposed to 1 mg/L of Hg, together with reduced phagocytic activity at 5 mg/L. Schaefer et al.^51^ also observed several changes in hematological parameters in wild bottlenose dolphins from Florida, USA. These findings underscore the immunosuppressive potential of the Hg in mammals, consistent with results observed in human cells *in vitro*.^61^ In addition to its immunotoxic effects, Hg has also been associated with chronic neurotoxic and hepatotoxic outcomes in aquatic mammals.^30^

Although it is not possible to establish a direct relationship between the observed effects and blood THg levels in the *I. geoffrensis* of this study, the hepatic concentrations reported by Lailson-Brito et al.^23^ suggest that some individuals from the Japurá River may be at risk of liver damage and pulmonary embolism.^57^ Future studies linking Hg concentrations in target organs, associated effects, and blood Hg levels would be valuable, as they may allow the estimation and monitoring of potential health effects using noninvasive sampling methods.

Like aquatic mammals, fish are one of the main vectors of contaminants to humans, as fishing is the main source of income and food for riverine populations.^62^ Assessing the health of top predators, such as *I. geoffrensis*, can be an effective way to extrapolate exposure scenarios to the local population, as the diet of this dolphin and humans is similar in several items.^33^ The use of *I. geoffrensis* as a sentinel is even more necessary in the region since the risk of human exposure to Hg via fish in the Amazon is critical, given the dietary habits of the communities there. Traditional communities (riverine, indigenous and quilombola) consume an average of 805 ± 1205 g (460–900 g) of fish per day.^63^ A recent systematic review of Hg in the main fish genera consumed by the Amazonian population reported mean Hg concentrations ranging from 80 to 500 μg/kg wet weight.^8^ Carnivorous fish from lakes, hydroelectric reservoirs, and blackwater ecosystems presented Hg concentrations in muscle above the thresholds in all studies, and, given the high fish consumption, health risks can be elevated even when Hg levels are below regulatory limits.^8^ The Solimões-Amazonas River and its tributaries provide most of the fish caught by commercial fisheries in the western Amazon, and Hg levels recorded in fish in these areas are above the recommended limits for many species.^8,62,64^ Therefore, monitoring the health of *I. geoffrensis* is necessary not only to conserve this endangered, endemic, and emblematic species of the Amazon but also to obtain an alert on the Hg exposure of the human populations, mainly riverine and indigenous communities.

### 3.2 Stable Isotope Measurements

The measurements of δ^13^C in the blood of *I. geoffrensis* ranged from −35.2‰ to −26.1‰, presenting a mean of −29.6 ± 1.7‰; whereas the δ^15^N mean was 9.8 ± 0.5‰, with values varying between 8.6‰ and 11.5‰ (Table 2, Figure 2). Considering the representativeness of the number of samples analyzed and the difficulty of obtaining them, this information is of great value in providing information on the species’ trophic position and foraging habits. The δ^13^C and δ^15^N values found in this study are in the same range as those reported for the muscles of *Cichla monoculus* (peacock bass) and *Pygocentrus nattereri* (piranha) from central Amazon.^65^ These species are also considered top predators in this aquatic ecosystem.

The δ^13^C values varied significantly between all sampling years, sexes, and age classes (Table S1), but all of them suggest the great importance of C3 primary producers in *I. geoffrensis* energy requirement. The 2004 (−29.1 ± 1.9‰) and 2006 (−30.6 ± 2.2‰) samplings differed significantly (*p* = 0.03). Differences were also found when comparing males (−29.1 ± 1.6‰) and females (−30.0 ± 1.7‰; *p* = 0.01). There was also a significant difference in the δ^13^C values between adults (−28.7 ± 1.3‰) and immature males (−31.4 ± 1.9‰) (*p* = 0.0007). Males showed higher δ^15^N values (9.9 ± 0.5‰) compared to females (9.6 ± 0.6‰) (*p* = 0.003). Regarding the temporal variation, only the δ^15^N values of 2004 (9.9 ± 0.6‰) were significantly different from 2011 (9.5 ± 0.4‰) (*p* = 0.04). No differences were found between age classes (Table S1).

Martin & da Silva^37^ reported habitat differences between males and females. Significant segregation was observed, with females and calves in the areas most remote from rivers, and males occupying the main river channel. However, at low water, an exodus from the floodplain to the river occurs and prevents the animals from being trapped.^37^ Since our samples were always collected during low water, at the junction of the Mamirauá Reserve entrance with the Japurá River, δ^15^N results suggest that males may be preying at higher trophic levels (larger prey). Besides, females use the *várzea* area more than males, an area with great prevalence of C4 plants (enriched in ^13^C),^66,67^ and we observed lower δ^13^C values in females compared to males. Indeed, although C4 macrophytes make up most of the primary producer biomass in the floodplains, the food web primarily relies on C3 carbon sources.^65,67^ Mortillaro et al.^65^ reported that in the central Amazon basin, there is a mismatch between the available food sources and those used by consumers.

Differences in the trophic ecology between males and females depend on species in cetaceans, reflecting a myriad of ecological and biological processes driven by sex. Therefore, spatial segregation, prey preferences, and even behavioral features can lead to some resource partition between sexes that is measurable by δ^13^C and δ^15^N.^35,68,69^ The present study found significant differences between the sexes, suggesting that the feeding ecology of male and female *I. geoffrensis* also differs. Considering this, KUD analysis was performed to better observe the niche distribution between males and females from different sampling years and to understand whether these animals show total or partial niche segregation (Figure 3).

For adults, differences were found in the isotopic niche width between sexes on the same year and in the same sex over the years (Figures S1 and S2, Table S2). Isotopic niche segregation occurs between males and females of *I. geoffrensis* in the MSDR (Figure 3). It is most evident in the 2004 samples, when the isotopic niche overlap between males and females was only approximately 30%. In 2011, the overlap increased to 40% to 60%, indicating partial segregation (Table 3). This segregation may result from the species’ behavior, with females and juveniles avoiding foraging in the same spaces as adult males, a behavior already reported by long-term monitoring.^37,70,71^ Indeed, our findings corroborate the sighting data from the field reported by Martin and da Silva.^37^ The authors described the sexual segregation in the population of *I. geoffrensis* of MSDR and attributed it to (i) differences in energetic requirements and (ii) the safety of females and/or calves from male harassment. Males are free to follow the females into the floodplain. However, the benefits of remaining on the main rivers seem to be greater, although still unclear. One hypothesis is the availability of larger-sized prey items. Indeed, males *I. geoffrensis* have been reported to exhibit high mobility, traveling long distances from the capture area, while females are known to be more allopatric, but both show a very strong site fidelity.^37^ Therefore, competition for prey and harassment from males toward calves and juveniles may be one of the factors explaining the niche segregation observed between males and females.^72^

The range of the isotopic niche decreased in 2011 compared to 2004 for both sexes. In 2004, even with greater resource use diversity, males and females exploited resources differently, i.e., there was less overlap. The results observed may suggest a lower availability of prey and/or use of the area in 2011. Despite this, major flood events, which could reduce the supply of prey, were not observed that year. Similarly, no extreme event occurred in 2004 or in the preceding years, with only a severe drought recorded in 2005.^15,73^ Males showed greater plasticity in their isotopic niche (Figure 3), mainly driven by δ^15^N values, suggesting changes in the trophic level of *I. geoffrensis* males over the years or changes in the signature in the base of the food web. Conversely, females show a high degree of niche overlap between years, indicating consistency in feeding strategies throughout the sampling period. However, the female samples in 2004 showed a wider isotopic area (Figure 3).

It is important to mention that although the accessible sampling design for the current study only includes the dry season, changes in foraging areas due to seasonal variations are expected. Significant changes in population density were observed between seasons, which are linked to known fish movements, which are, in turn, dictated by changes in water level and dissolved oxygen.^37^ Thus, future studies evaluating the seasonal variations in the isotopic niche of *I. geoffrensis* may provide more interesting findings.

Additionally, the construction of dams and hydroelectric plants in the region alters the flood pulse of the rivers in the Amazon basin.^38,74^ This infrastructure not only changes the cycle of contaminants,^75,76^ as mentioned above, but can also alter the richness, distribution, and abundance of species in the ecosystem, causing economic and social problems in the region, as well as species conservation.^2,14,74^ In the case of *I. geoffrensis*, hydroelectric plants and dams fragment the habitat, isolating populations and reducing the gene pool, increasing the likelihood of local extinctions.^16^ They also affect the abundance and presence of prey, resulting in reduced dietary diversity.^18,20^ As shown in the present study, *I. geoffrensis* populations exhibit niche segregation that, if altered, may increase intraspecific competition, mainly affecting the survival of females and calves.

### 3.3 Generalized Linear Model

A Generalized Linear Model was used to understand the influence of ecological and biological features on THg concentrations in *I. geoffrensis*. The model that best explained the THg concentrations of blood samples of *I. geoffrensis* from the MSDR included the following variables: sex, total length (TL), δ^13^C, and δ^15^N. The δ^13^C (*p* = 0.01) was the significant variable, while δ^15^N (*p* = 0.05) and total length (*p* = 0.05) were marginally significant variables (Table S3). This model also explained the variation in THg concentration (pseudo-*R* ^2^ = 0.37) (Figure 4), highlighting higher THg concentrations in males than in females (Figure S3).

Regardless of sex, δ^13^C values influence Hg levels, indicating that the foraging area is a key driver of Hg exposure in these animals (Figure 4). This pattern might reflect differences between the food webs of the *várzea* and the main river channel, representing a gradient in the contribution of terrigenous versus riverine carbon sources. Carbon sources are known to influence Hg availability in different ecosystems, and two key factors have been identified in the Amazon:^74,76^ (i) aquatic environments promote Hg methylation and ionizing^75^ and (ii) phytoplankton, which is more readily degraded than plant debris, enhances Hg methylation.^76^ Another well-established driver of Hg exposure is trophic level, as reflected by δ^15^N values. Despite the marginal significance found (*p* = 0.05), which might be an artifact of the narrow δ^15^N range observed in data set,^77^ the effect size was meaningful (η^2^ = 0.09). Mercury exposure was also slightly associated with total body length, with larger individuals having higher Hg concentrations (*p* = 0.05). Larger animals have greater energetic demands, which leads to increased food intake and, consequently, higher Hg exposure.

**Figure 2:**
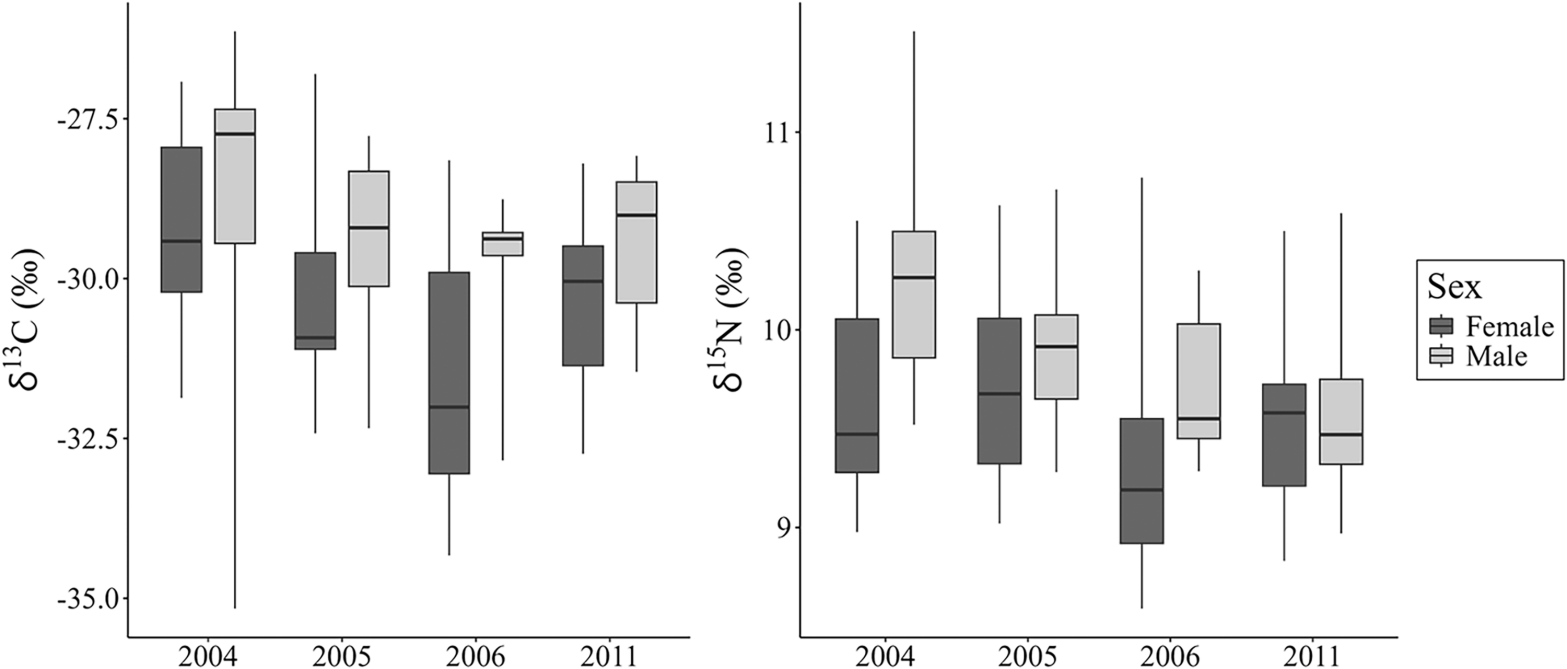
Measurements of δ^13^C (left) and δ^15^N (right) in blood samples from female and male of Amazon River dolphins (*Inia geoffrensis*) collected between 2004 and 2006 and 2011 in the Mamirauá Sustainable Development Reserve, Brazilian Amazon.

**Table 2:** Measurements of δ^13^C and δ^15^N (‰) in Blood Samples of Amazon River Dolphin (*Inia geoffrensis*) Collected between 2004 and 2006 and 2011 in the Mamirauá Sustainable Development Reserve, Brazilian Amazon

		Adult				Immature				All			
Year	Sex	*N*	Mean ± SD	Median	Min/Max	*N*	Mean ± SD	Median	Min/Max	*N*	Mean ± SD	Median	Min/Max
δ^13^C (‰)													
2002	Female	1	-	-	–30.2	na	na	na	na	1	-	-	–30.2
	Male	1	-	-	–28.8	na	na	na	na	1	-	-	–28.8
2003	Female	na	na	na	na	na	na	na	na	na	na	na	na
	Male	2	–28.2 ± 0.9	–28.2	–28.8/–27.5	na	na	na	na	2	–28.2 ± 0.9	–28.2	–28.8/–27.5
2004	Female	17	–29.1 ± 1.5	–29.3	–31.9/–26.9	2	–30.7 ± 1.3	–30.7	–31.6/–29.8	19	–29.3 ± 1.5	–29.4	–31.9/–26.9
	Male	14	–27.9 ± 1.3	–27.7	–30.8/–26.1	2	–33.0 ± 3.1	–33.0	–35.2/–30.8	16	–28.5 ± 2.3	–27.7	–35.2/–26.1
2005	Female	5	–30.1 ± 2.2	–31.1	–32.4/–26.8	1	–30.8	–30.8	–30.8	6	–30.2 ± 2.0	–30.9	–32.4/–26.8
	Male	18	–29.0 ± 1.0	–28.9	–31.6/–27.8	4	–31.2 ± 0.9	–31.0	–32.3/–30.4	22	–29.4 ± 1.3	–29.2	–32.3/–27.8
2006	Female	6	–31.5 ± 2.4	–32.0	–34.3/–28.2	0	na	na	na	6	–31.5 ± 2.4	–32.0	–34.3/–28.2
	Male	4	–29.3 ± 0.4	–29.3	–29.6/–28.8	1	–32.8	–32.8	–32.8	5	–30.0 ± 1.6	–29.4	–32.8/–28.8
2011	Female	13	–30.5 ± 1.3	–30.1	–32.7/–28.2	3	–29.7 ± 1.5	–29.1	–31.4/–28.7	16	–30.3 ± 1.3	–30.0	–32.7/–28.2
	Male	9	–28.8 ± 0.8	–28.5	–30.4/–28.1	8	–30.0 ± 1.1	–30.2	–31.5/–28.2	17	–29.4 ± 1.1	–29.0	–31.5/–28.1
δ^15^N (‰)													
2002	Female	1	10.8	10.8	10.8	na	na	na	na	1	10.8	10.8	10.8
	Male	1	9.7	9.7	9.7	na	na	na	na	1	9.7	9.7	9.7
2003	Female	na	na	na	na	na	na	na	na	na	na	na	na
	Male	2	10.2 ± 0.5	10.2	9.9/10.6	na	na	na	na	2	10.2 ± 0.5	10.2	9.9/10.6
2004	Female	17	9.7 ± 0.5	9.5	9.0/10.6	2	9.1 ± 0.2	9.1	9.0/9.3	19	9.6 ± 0.5	9.5	9.0/10.6
	Male	14	10.3 ± 0.6	10.3	9.5/11.5	2	9.9 ± 0.5	9.9	9.6/10.3	16	10.3 ± 0.6	10.3	9.5/11.5
2005	Female	5	9.6 ± 0.4	9.5	9.0/10.1	1	10.6	10.6	10.6	6	9.7 ± 0.6	9.7	9.0/10.6
	Male	18	9.9 ± 0.3	9.9	9.3/10.4	4	9.8 ± 0.6	9.6	9.3/10.7	22	9.9 ± 0.4	9.9	9.3/10.7
2006	Female	6	9.4 ± 0.8	9.2	8.6/10.8	0	na	na	na	6	9.4 ± 0.8	9.2	8.6/10.8
	Male	4	9.8 ± 0.4	9.8	9.5/10.3	1	9.3	9.3	9.3	5	9.7 ± 0.4	9.6	9.3/10.3
2011	Female	13	9.4 ± 0.4	9.4	8.8/10.0	3	10.1 ± 0.4	10.0	9.6/10.5	16	9.5 ± 0.4	9.6	8.8/10.5
	Male	9	9.5 ± 0.3	9.5	9.1/10.0	8	9.5 ± 0.5	9.4	9.0/10.6	17	9.5 ± 0.4	9.5	9.0/10.6

**Figure 3:**
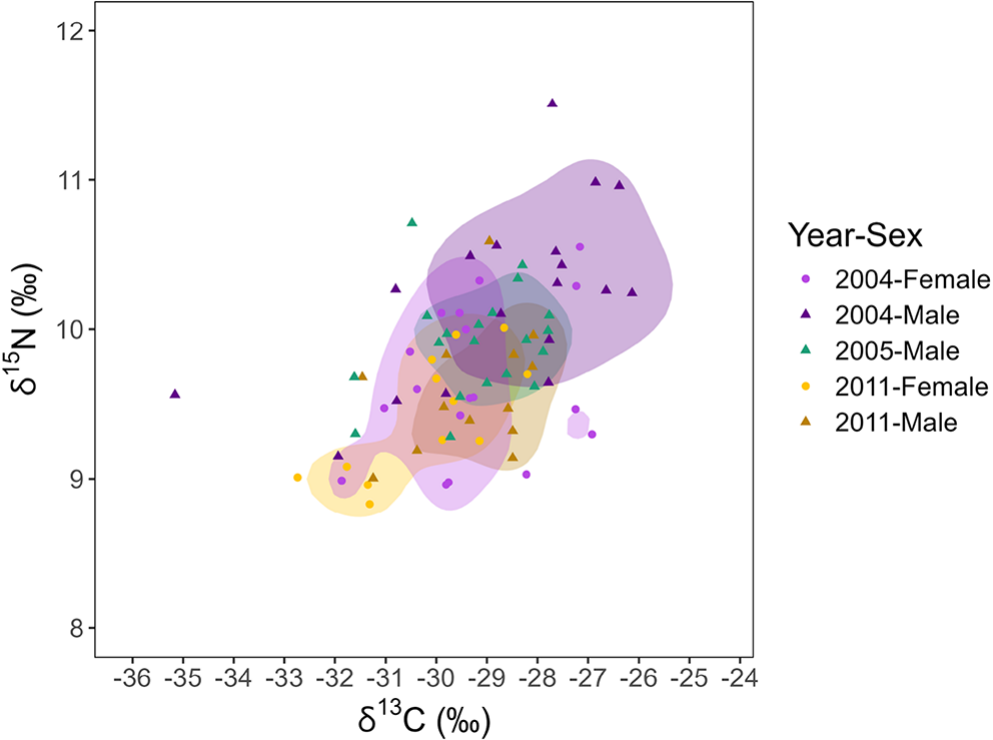
Kernell niche density analysis of female and male individuals of Amazon River dolphins (*Inia geoffrensis*) from the Mamirauá Sustainable Development Reserve, Brazilian Amazon. Samples were collected in different years (2004, 2005, and 2011).

**Table 3:** Isotopic Niche Overlap (%) Estimative for Amazon River Dolphin (*Inia geoffrensis*) by Year and Sex According to Kernel Utilization Densities at 50%, 75%, and 90% Contours^a^

		50%	75%	90%
2011	Female × Male	46.4	66.5	71.6
	Male × Female	57.4	75.6	79.8
2004	Female × Male	25.5	64.7	78.1
	Male × Female	18.3	45.2	49.1
Male	2011 × 2005	50.1	60.8	73.2
	2011 × 2004	39.0	73.8	87.8
	2005 × 2011	58.5	67.1	77.6
	2004 × 2011	17.0	30.6	36.2
	2005 × 2004	76.6	98.1	99.7
	2004 × 2005	28.6	38.2	38.8
Female	2011 × 2004	39.0	83.2	88.1
	2004 × 2011	58.5	58.2	64.5

a Overlaps are the percentage of the first group area over the second.

**Figure 4:**
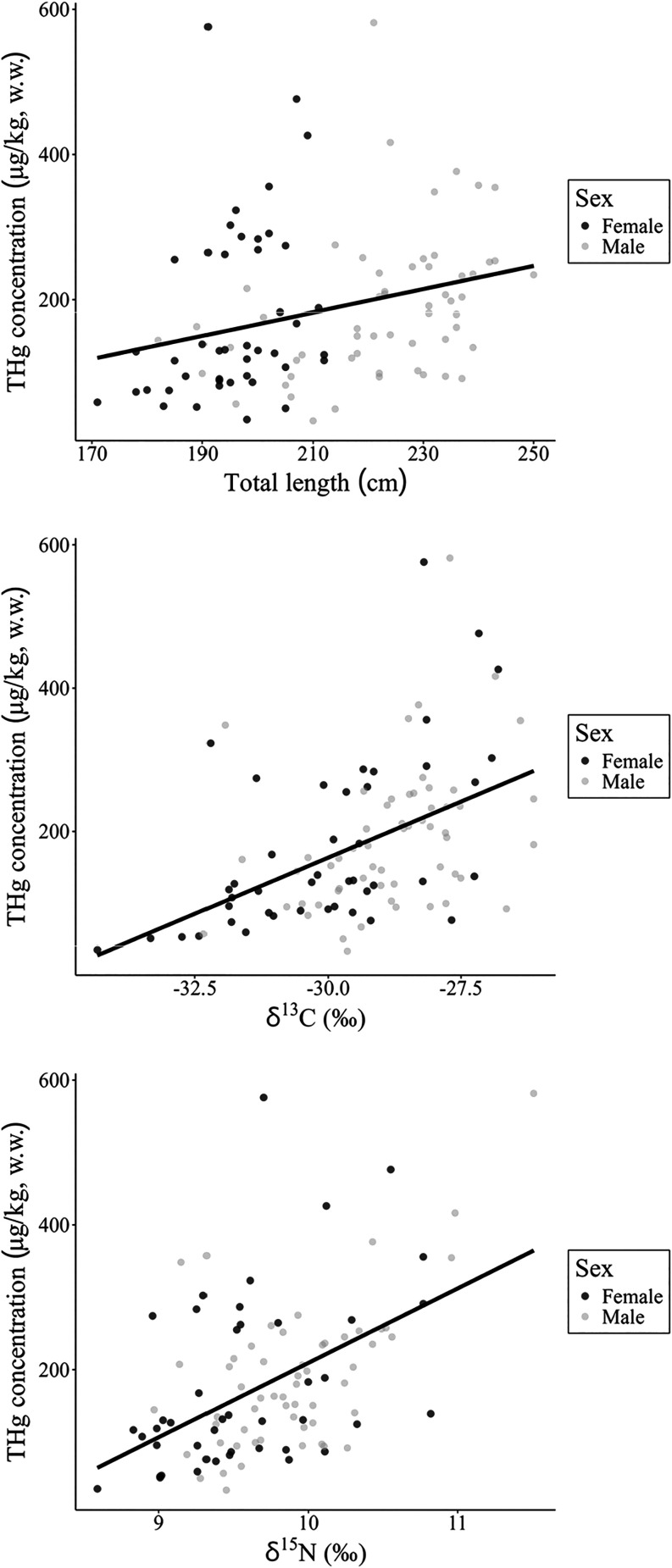
Total mercury concentrations (μg/kg, wet weight) in Amazon River dolphins (*Inia geoffrensis*) blood samples are explained by total length (cm), δ^13^C and δ^15^N values and sex, these being the significant variables in the generalized linear model (GLM).

The spatial segregation that occurs between males and females of *Inia geoffrensis* in the MSDR seems to be the main factor behind the differences observed in THg concentrations, which are explained by the δ^13^C and δ^15^N values. The findings elucidate the susceptibility of different specimens to Hg exposure, with adult males showing higher levels of this toxic metal. This information is critical for informing conservation strategies.

### 3.4 The Future: Climate Change

Global greenhouse gas emissions and deforestation in the Amazon have contributed to climate change, resulting in rising temperatures, changes in precipitation patterns and more frequent and intense extreme events. In recent decades, the Amazon has experienced severe droughts in 1997, 2005, and 2010, and major floods in 2009, 2012, 2013, and 2014.^15^ As a consequence, the flood pulse—a key driver of aquatic ecosystem dynamics—has been disrupted, affecting all the ecological processes it regulates.^15,78,79^

Climate change is predicted to exacerbate Hg methylation in aquatic ecosystems,^80^ which is a major concern due to the toxicity of MeHg. Additionally, the exposure of *I. geoffrensis* to Hg through their prey will be exacerbated.^80^ Rising temperatures will promote an accelerated metabolism in fish and other aquatic species, resulting in smaller individuals, i.e., smaller prey. For top predators such as *I. geoffrensis*, this means eating a greater amount (in number) of fish, resulting in greater Hg exposure.^80^ Combined with other stressors such as malnutrition, Hg stored in accumulation organs can be remobilized into the bloodstream, becoming available to cause adverse effects in target organs, as has already been reported in marine mammals.^81^

Specifically for dolphins, drought and rising temperatures have led to thermal stress that has killed hundreds of individuals during recent years of extreme drought.^13,14^ These droughts will lead to habitat loss, resulting in greater niche overlap between individuals and species. Given that the observed niche segregation for the species is driven by sex and age class, this may increase the competition between males and females and immature individuals. Due to their smaller size and habit of foraging on riverbanks and floodplains, females and juveniles will likely suffer more pressure in this scenario. These biogeochemical and ecological perturbations, therefore, highlight the importance of incorporating climate-induced variability into conservation strategies for Amazonian aquatic ecosystems.

*I. geoffrensis* in this study showed significant Hg levels in their blood, indicating recent exposure, and making Hg a matter of concern even within protected conservation areas such as the MSDR. In addition, the integrated analysis of historical contaminants and stable isotopes provides valuable insights into Hg exposure and the intraspecific ecological characteristics of this species. When combined with habitat loss, this scenario of high bioavailability of Hg in the region poses even greater threats to this iconic Amazonian species.

## Supplementary Material



## Notes

Figure S1. Estimates of isotopic niche area overlap of females *Inia geoffrensis*; Figure S2. Estimates of isotopic niche area overlap of males *Inia geoffrensis*; Figure S3. Mercury concentrations (μg/kg, wet weight) in blood samples; Table S1. ANOVA values for δ^13^C and δ^15^N in blood samples; Table S2. Isotopic niche areas; Table S3. Results of the Generalized Linear Model (GLM) (PDF)
