# Supplementary material for: Elucidating Sexual and Spatial Influences on the Trophic Ecology of Amazon River Dolphins (Inia geoffrensis) and Mercury Contamination
Source: Environ Sci Technol. 2025 Oct 16;59(42):22452–64. doi: 10.1021/acs.est.5c07115 (PMC12573784; doi:10.1021/acs.est.5c07115)
Supplement: Supplementary file 1 [file es5c07115_si_001.pdf]

Supporting Information

Elucidating sexual and spatial influences on the trophic ecology of Amazon River dolphins (*Inia geoffrensis*) and mercury contamination

Monizze Vannuci-Silva[1,2*]; Vera Maria Ferreira da Silva[3]; Lucas Rodrigues Tovar[1]; Rodrigo de Souza Amaral[4]; Bárbara M. R. Manhães[1]; Gleici Montanini[1]; Alexandre de Freitas Azevedo[1,2]; José Lailson-Brito[1,2]; Tatiana Lemos Bisi[1,2]

This file contains:

- Total pages: 4
- Figures: 3
- Tables: 3

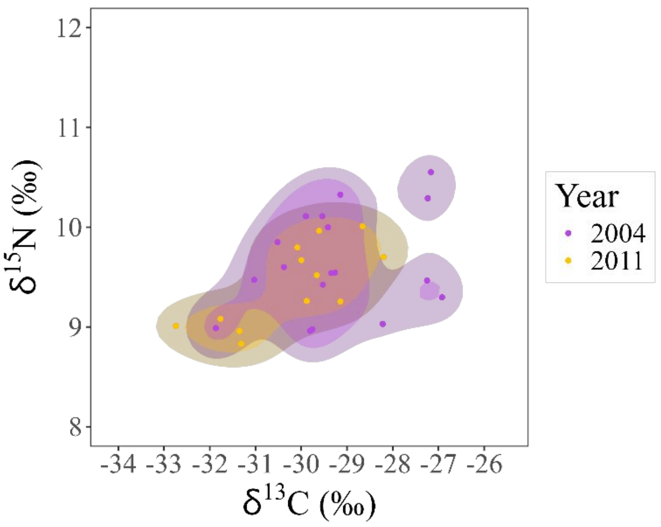

Figure S1. Estimates of isotopic niche area overlap of females *Inia geoffrensis* from the Mamirauá Sustainable Development Reserve, Brazilian Amazon, sampled in different years (2004 and 2011) using kernel utilization density estimators at 50% and 75% contours.

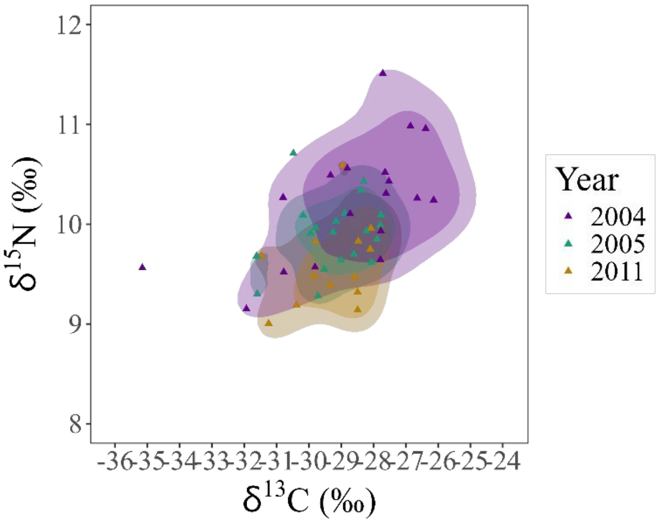

Figure S2. Estimates of isotopic niche area overlap of males *Inia geoffrensis* from the Mamirauá Sustainable Development Reserve, Brazilian Amazon, sampled in different years (2004, 2005, and 2011) using kernel utilization density estimators at 50% and 75% contours.

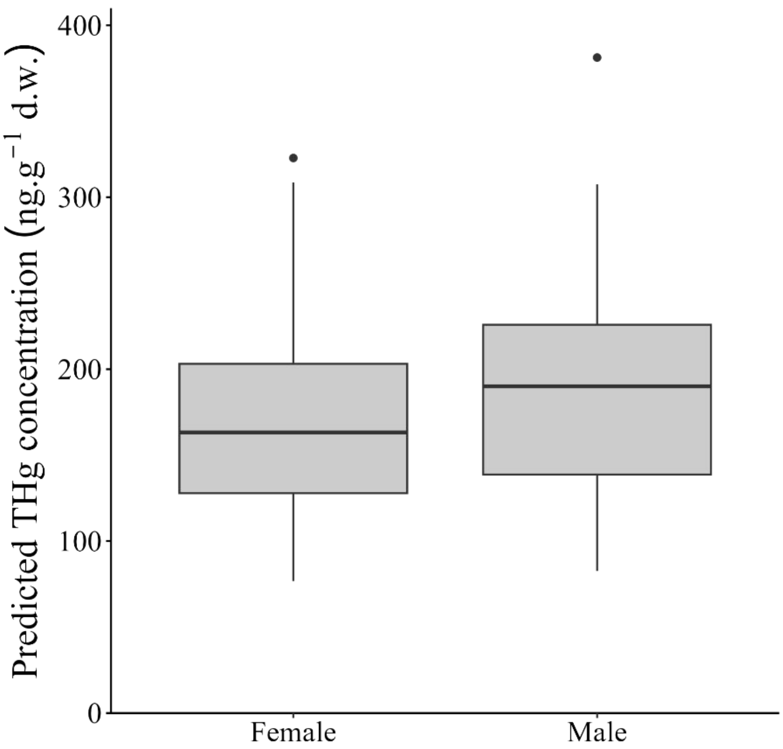

Figure S3. Predicted total mercury concentrations (µg/kg, wet weight) in blood samples of females and males of *Inia geoffrensis* from the Mamirauá Sustainable Development Reserve, Brazilian Amazon, using the best-fitted GLM model. Considering the concentrations predicted by the model, the values in females and males are significantly different.

Table S1. ANOVA values for $\delta^{13}C$ and $\delta^{15}N$ in blood samples of Amazon River dolphin (*Inia geoffrensis*) individuals sampled between 2004-2006 and 2011 in the Mamirauá Sustainable Development Reserve, Brazilian Amazon. F=Fischer test; p= p value; η^2= effect size.

	$\delta^{13}C$			$\delta^{15}N$		
	F	p	η^2	F	p	η^2
Year	2.49	0.04	0.12	3.03	0.01	0.14
Sex	9.95	0.01	0.10	9.19	0.01	0.09
Age class	4.87	0.01	0.13	1.50	0.22	0.05
Year: Sex	0.23	0.92	0.01	2.99	0.02	0.11
Year: Age class	1.16	0.33	0.08	1.92	0.07	0.13
Sex: Age class	4.47	0.01	0.09	0.45	0.64	0.01
Year: Sex: Age class	0.74	0.48	0.02	1.34	0.27	0.03

Table S2. Isotopic niche areas considering the confidence interval of 50%, 75% and 90% for females and males of Amazon River dolphins (*Inia geoffrensis*) collected in 2004 (females=17; males=14), 2005 (males=18) and 2011(females=13; males=9) from the Mamirauá Sustainable Development Reserve, Brazilian Amazon.

Year	Confidence interval (%)	Area (‰^{2})	
		Female	Male
2004	50	3.79	5.28
	75	7.6	1.09
	90	1.12	1.79
2005	50	na*	1.97
	75	na*	4.24
	90	na*	6.97
2011	50	2.85	2.3
	75	5.32	4.67
	90	8.23	7.38

*na= not available

Table S3. Results of the Generalized Linear Model (GLM) for the total mercury (THg) concentrations in blood samples of Amazon River dolphins (*Inia geoffrensis*) collected between 2004 and 2006, and 2011 in the Mamirauá Sustainable Development Reserve, Brazilian Amazon. β= model intercept; SE= standard error; t= t test; p= p value; pseudo η^2= effect size value.

	Estimate	SE	t	p	pseudo η^2
β	4.70	2.30	2.04	0.04	-
$\delta^{13}C$	0.12	0.04	2.92	0.01	0.25
Total length	0.01	0.01	2.01	0.05	0.01
$\delta^{15}N$	0.25	0.13	1.95	0.05	0.09
Sex	-0.34	0.15	-2.27	0.02	0.05
